# Economic Evaluation of First-Line Atezolizumab for Extensive-Stage Small-Cell Lung Cancer in the US

**DOI:** 10.3389/fpubh.2021.650392

**Published:** 2021-04-06

**Authors:** Yingcheng Wang, Mingjun Rui, Lan Yang, Xintian Wang, Ye Shang, Aixia Ma, Hongchao Li

**Affiliations:** ^1^School of International Pharmaceutical Business, China Pharmaceutical University, Nanjing, China; ^2^Center for Pharmacoeconomics and Outcomes Research, China Pharmaceutical University, Nanjing, China

**Keywords:** small-cell lung cancer, cost-effectiveness, atezolizumab, chemotherapy, extensive stage

## Abstract

**Introduction:** This study evaluated the cost-effectiveness of atezolizumab + chemotherapy vs. chemotherapy as first-line treatment for extensive-stage small-cell lung cancer (SCLC) in the United States (US).

**Methods:** The three health states partitioned survival (PS) model was used over the lifetime. Effectiveness and safety data were derived from the IMpower133 trial. The parametric survival model and mixture cure model were used for the atezolizumab + chemotherapy group to explore the long-term uncertainty of the effect of immunotherapy, and the parametric survival model was used for the chemotherapy group. Costs were derived from the pricing files of Medicare and Medicaid Services, and utility values were derived from previous studies. Sensitivity analyses were performed to observe model stability.

**Results:** If the mixture cure model was considered for the intervention group, compared with chemotherapy alone, atezolizumab + chemotherapy yielded an additional 0.11 quality-adjusted life-years (QALYs), with an incremental cost of US$84,257. The incremental cost-utility ratio (ICUR) was US$785,848/QALY. If the parametric survival model was considered for the intervention group, atezolizumab + chemotherapy yielded an additional 0.10 QALYs, with an incremental cost of US$84,257; the ICUR was US$827,610/QALY. In the one-way sensitivity analysis, progression-free (PF) and postprogression (PP) utilities were the main drivers. In the scenario analysis (PF utility = 0.673, PP utility = 0.473), the results showed that the ICUR was US$910,557/QALY and US$965,607/QALY when the mixture cure model and parametric survival model was considered for the intervention group, respectively. In the PSA, the probabilities that atezolizumab + chemotherapy would not be cost-effective were 100% if the willingness-to-pay threshold was US$100,000/QALY.

**Conclusions:** The findings of the present analysis suggest that atezolizumab + chemotherapy is not cost-effective in patients receiving first-line treatment for extensive-stage SCLC in the US.

## Introduction

Lung cancer is currently the second most common cancer classified by sex; after prostate cancer in men and breast cancer in women, lung cancer accounts for 14% of new cancers in American men and 13% of new cancers in American women (1). Extensive-stage small-cell lung cancer (SCLC) accounts for ~15% of all lung cancers (2). The prognosis of SCLC is usually poor. It has been reported that the median overall survival (OS) of patients with SCLC is 8–12 months, and the annual OS rate is ~5%. Three first-line treatment options are currently recommended for extensive-stage SCLC: (1) carboplatin + etoposide + atezolizumab, (2) carboplatin + etoposide + durvalumab, and (3) cisplatin + etoposide + durvalumab (3).

Atezolizumab is a humanized monoclonal antibody designed to bind to the PD-L1 protein expressed on tumor cells and tumor-infiltrating immune cells, blocking its interaction with the PD-1 and PD-L1–B7- 1 receptors (4). By inhibiting PD-L1, atezolizumab can activate T cells, allowing them to kill cancer cells. Atezolizumab was approved by the Food and Drug Administration FDA for the first-line treatment of SCLC, based on the IMpower133 trial (NCT02763579), which was a randomized, multicenter, double-blind, placebo-controlled phase III trial (5). This trial studied 403 patients who had not previously received chemotherapy and had an Eastern Cooperative Oncology Group (ECOG) performance status score of 0 or 1. The patients were randomly divided into two groups: the intervention group received carboplatin + etoposide + atezolizumab, and the control group received carboplatin + etoposide + placebo. The primary outcomes were OS and progression-free survival (PFS).

The trial showed that atezolizumab combined with chemotherapy could significantly prolong survival time [median OS = 12.3 vs. 10.3 months; hazard ratio (HR) = 0.70, 95% confidence interval (CI): 0.54–0.91; *p* = 0.0069], which meant that the risk of death was reduced by 30%, and patients receiving atezolizumab were more likely to survive within 1 year (1-year survival rate = 51.7%) than patients in the control group (1-year survival rate = 38.2%). Compared with chemotherapy alone, the median PFS of patients receiving atezolizumab combined with chemotherapy also improved (median PFS = 5.2 vs. 4.3 months in the placebo group; HR = 0.77; 95% CI: 0.62–0.96; *p* = 0.017), significantly reducing the risk of disease deterioration or death. Serious adverse reactions occurred in 37% of patients receiving atezolizumab combined with chemotherapy, compared with 35% of patients receiving chemotherapy alone. Although the addition of atezolizumab to chemotherapy resulted in significantly clinical efficacy, the immunotherapy causes enormous medical expenditure which brings economic burden to patients and governments. According to the statistics, biologic therapies are among the most expensive drugs, accounting for 40% of total US spending on prescription drugs despite only 2% of patients using biologics (6). Therefore, the economic evaluation is required to explore the clinical effectiveness and the cost-effectiveness of the new intervention showing whether it is available to patients.

In the economic evaluation of tumor drugs, we usually need to fit the curves from the clinical trials to construct a Markov model or a partitioned survival (PS) model (7). Even when the data for OS and PFS curves are immature, we still need to extrapolate them based on the fit results. In many previously published studies, standard parametric models were used. The commonly used fitting distributions include the exponential distribution, Weibull distribution, Gompertz distribution, lognormal distribution, etc. The reason for the selection of a parameter distribution was explained in some (8–10) but not all studies (11). However, many researchers overlooked a problem: the premise for the application of the standard parametric model was that all observed subjects would have an expected event at a certain point in time within a defined sufficient follow-up time. Obviously, this would not be suitable for data analysis of long-term survival analyses. Some immunotherapies, such as PD-1/PD-L1 or CAR-T, might keep the patient alive for a longer time or even cure the patient; therefore, the Kaplan–Meier (KM) curve showed an obvious plateau. For this case, the mixed cure model was also an important alternative method in addition to the standard parameter model, and it would be more suitable under certain circumstances. We can also see that an increasing number of studies are trying to use the mixture parametric model instead of the standard parametric model (12, 13). For example, Roth et al. (12) used a cure model to estimate the proportion achieving long-term survival.

Information on the cost-effectiveness of immunotherapy PD-L1 is required by decisionmakers in the health care system to determine the value of these novel treatment in extensive-stage SCLC in the world, for example NICE had already released Technology appraisal guidance [TA638] (https://www.nice.org.uk/guidance/ta638). The objective of this study was to explore the cost-effectiveness of atezolizumab in the United States (US). Besides, our research also explored how the use of the standard parametric model vs. the mixture parametric model would affect the incremental cost-utility ratio (ICUR) and incremental cost-effectiveness ratio (ICER) results.

## Methods

### Patient Cohort and Treatment Duration

The patients included in the cohort were those who were adults with extensive-stage SCLC who had not previously received chemotherapy, which was consistent with the IMpower133 trial (5). We assumed that all patients were in the progression-free (PF) health state, and they received one of the following two treatments at the beginning of the model:

1. The patients in the intervention group received four 21-day cycles of carboplatin (area under the curve of 5 mg per milliliter per minute) and etoposide (100 mg per square meter of body surface area). In addition, they received atezolizumab (1,200 mg) in each 21-day cycle until disease progression or death.
2. The patients in the control group received four 21-day cycles of carboplatin (area under the curve of 5 mg per milliliter per minute) and etoposide (100 mg per square meter of body surface area).

### Model Overview

**Figure 1:**
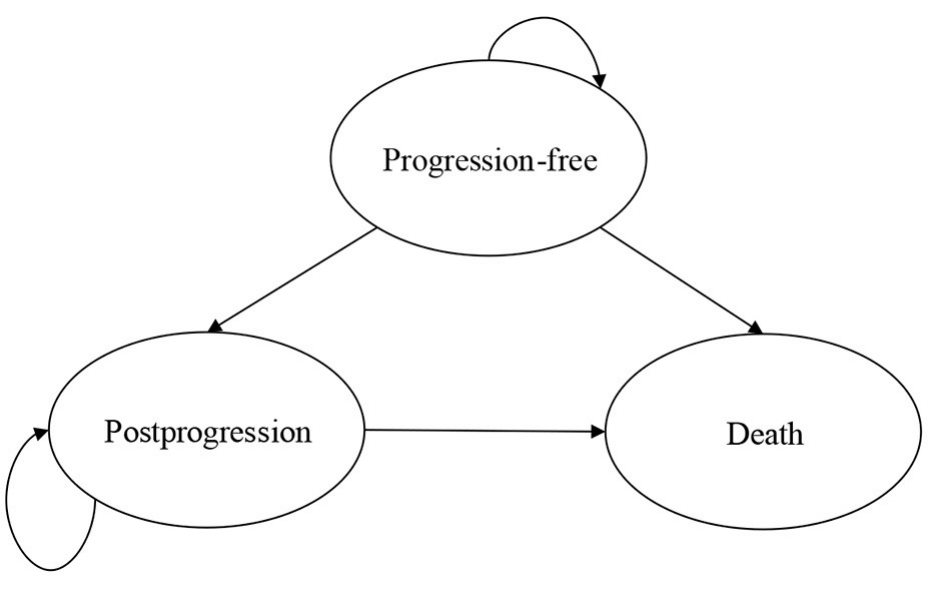
Model structure.

A PS model with three mutually exclusive health states was developed to estimate the costs and outcomes of the first-line treatment of patients with extensive-stage SCLC in Microsoft Excel (Figure 1). The proportion of patients in the postprogression (PP) state in each cycle was calculated as the difference between OS and PFS based on data from the IMpower133 trial. Each cycle lasted 3 weeks, which was consistent with the IMpower133 trial. A lifetime horizon (2.5 years) was chosen, and ≥95% of patients in the control group died. We examined the following outcomes: quality-adjusted life-years (QALYs), life-years gained (LYGs), and costs. All of them were discounted by 3%. The US payer perspective was taken; therefore, only the direct medical care costs were included.

### Model Approaches

#### Standard Parametric Models

There are a wide range of parametric models available, and each have their own characteristics which make them suitable for different data sets. Standard models considered in our studies were the proportional hazards-based exponential, Weibull, Gompertz, and the accelerated failure time-based log-normal, log-logistic. In fact, the standard models have been commonly used in National Institute for Health and Care Excellence appraisals (14).

#### Cure Models

This model was first proposed by Boag et al. in 1949 (15). Their study showed that in the survival analysis of tumor data, the existence of “long-term survival” needed to be considered. The key to the cure model is to determine how many patients are “cured,” that is, there exists a long-term survival situation in them (16). In this model, Logistic regression and Probit regression are usually used to predict whether the patient is “cured” (17). The death risk of cured patients is generally assumed to be similar to the background population, while the death risk of uncured patients is the mortality of the diseased population.

There are currently two main types of cure models: mixture cure model and non-mixture cure model. In the mixture cure model, the population is divided into two groups, namely the uncured group and the cured group, and the population is analyzed as the mixed distribution of the two groups. In contrast, it is assumed that all patients are in the same group in the non-mixture cure model, but the risk of the event decreases to 0 over time changes over time.

The survival function of the mixture cure model can be written as shown in Equation (1) (18):

(1) $$\begin{array}{rc} \text{Surviva}{\text{l}}_{\text{population}} & =\text{Surviva}{\text{l}}_{\text{generalpopulation}}*({\text{p}}_{\text{cured}}+\\ {\left(1-{\text{p}}_{\text{cured}}\right)}^{*}\text{Surviva}{\text{l}}_{\text{uncured}}) \end{array}$$

The survival function of the mixture cure model can be written as shown in Equation (2) (18):

(2) $$\begin{array}{rc} \text{Surviva}{\text{l}}_{\text{population}} & =\text{Surviva}{\text{l}}_{\text{generalpopulation}}*\text{exp}(\text{Ln}\\ {\left({\text{p}}_{\text{cured}}\right)}^{*}\left(1-\text{S}\right)) \end{array}$$

S is a standard extrapolation distribution that decreases to 0 over time

It should be noted that the premise of non-mixture cure model is: survival data should meet the proportional hazard assumption, because the non-mixture cure model is a proportional hazards model of cure (19).

### Overall Survival and Progression-Free Survival

Long-term OS and PFS were estimated by fitting parametric survival distributions to pseudo-individual participant data (IPD) derived from digitized KM curves from the IMpower133 trial, and the pseudo-IPD data were extracted with Engauge Digitizer software (http://markummitchell.github.io/engauge-digitizer/). To extrapolate the probability of survival to cover the lifetime horizon, we considered five standard parametric models in Microsoft Excel to fit two curves: exponential distribution, the Weibull distribution, the Gompertz distribution, loglogistic distribution, and lognormal distribution. For the special mechanism of PD-L1 and the obvious plateau at the end of the curve, the mixture cure model was also considered for the intervention group (carboplatin + etoposide + atezolizumab), which was conducted in R 3.6.0. The OS and PFS curves were found to be inconsistent with the proportional hazard (PH) assumption; therefore, the non-mixture cure model was not considered (20). Parametric survival models and mixture cure models were selected based on Akaike's information criterion (AIC) statistic and visual inspection of survival distributions. The lowest AIC values were considered as goodness-of-fit statistics, and visual inspection was to check whether the distribution was fit to KM curve. External data were also used to validate the long-term extrapolations of the control group (21). When few data were available about long-term extrapolations of atezolizumab in SCLC, two kinds of models (standard parametric models and mixture cure models) were used to extrapolate the intervention group, which is explained in the discussion section. The Gompertz distribution was used to estimate OS for the atezolizumab + chemotherapy group and chemotherapy group based on its good statistical and visual inspection. For the mixture model, the Gompertz (restricted) distribution was used to estimate OS for the atezolizumab + chemotherapy group based on its good statistical and visual fit.

**Figure 2:**
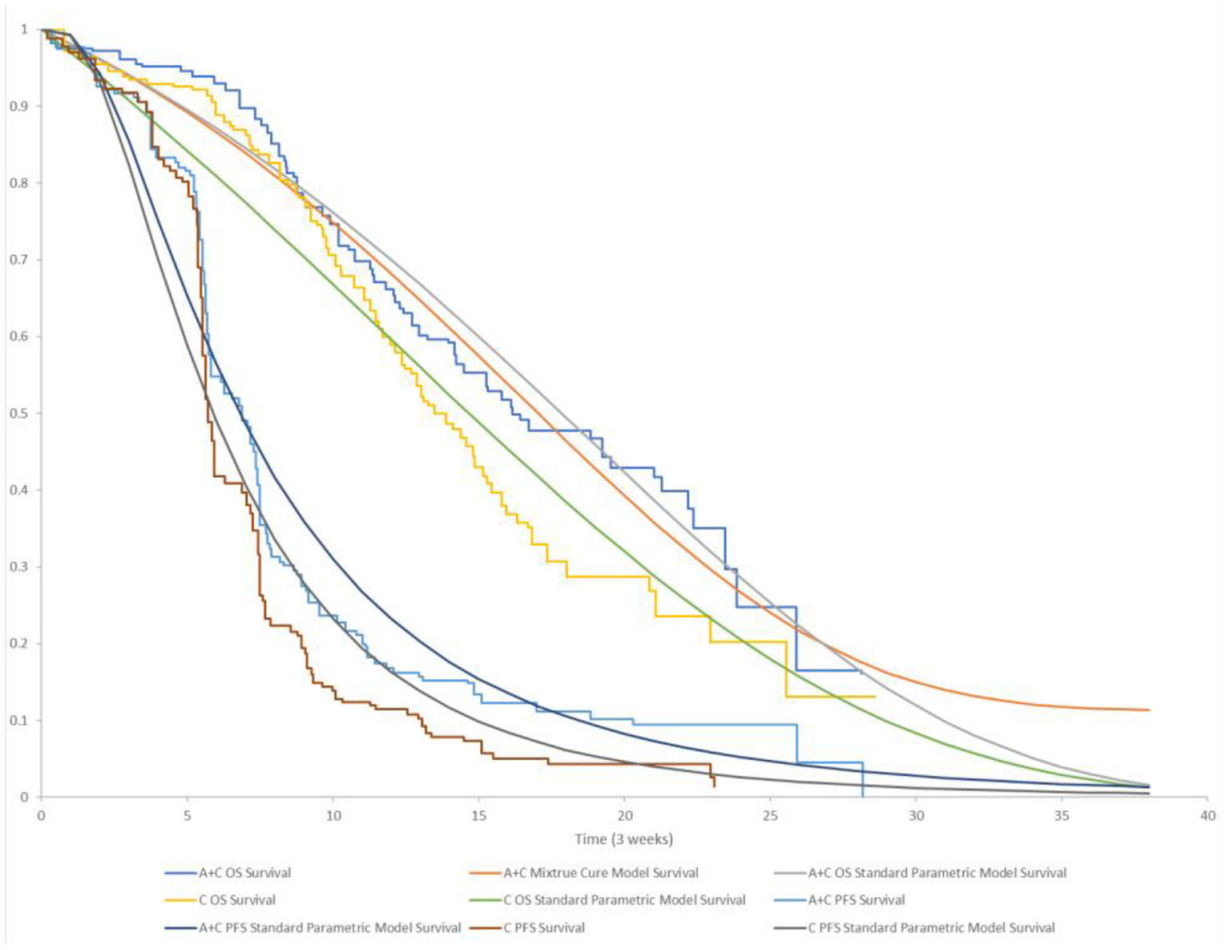
KM and parametric survival distributions for OS and PFS. A + C, atezolizumab + chemotherapy; C, chemotherapy.

PFS curves were estimated based on the lognormal distribution fit to pseudo-IPD. Age- and sex-adjusted US general population mortality rates were additively applied to the estimated parametric survival distributions for PFS and OS to account for natural mortality, which was referred to background population in the cure model (22). While the death risk of the diseased population was derived from the OS curve. KM and parametric survival distributions for OS and PFS used in the model are shown in Figure 2. Additional details regarding the selection of parametric survival models and mixture cure models are provided in the Supplementary Material.

### Utility Values

Because little information on the quality of life of SCLC patients was collected in the IMpower133 trial and other published studies, utilities of each health state were obtained from previously published studies about NSCLC (0.840 for PF, 0.473 for PP and 0 for death) (23).

Disutilities for adverse events (AEs) whose values were taken from published sources were also included for patients receiving treatment (i.e., atezolizumab + chemotherapy and chemotherapy) (23–25). The duration of AEs was derived from the published clinical expert opinion (10).

### Cost

Direct medical costs include drugs, administration, and costs for the management of adverse reactions. We found that in the IMpower133 trial, ~40.3% of patients in the atezolizumab + chemotherapy group received subsequent chemotherapy, but the trial did not describe detailed second-line treatment options; therefore, we consequently assumed that 40.3% of patients who progressed while receiving atezolizumab + chemotherapy group received topotecan (0.1 mg, at 1.5 mg/m^2^ daily on days 1–5 of each 21-day cycle) and 15.4% of patients received CAV (cyclophosphamide, 25 mg, 1,000 mg/m^2^ on day 1; doxorubicin, 10 mg, 45.0 mg/m^2^ on day 1; vincristine, 1 mg, 2.0 mg on day 1 of each 21-day cycle) (26) as their second-line treatment according to the National Comprehensive Cancer Network (NCCN) guideline (27). Similarly, we assumed that 43.6% of patients who progressed while receiving chemotherapy received topotecan and 22.8% of patients received CAV as their second-line treatment. A body weight of 70 kg, a body surface area of 1.86 m^2^, and a creatinine clearance of 70 mL/min were assumed in the model from the published literature (28). The 2019 average sale price from the Centers for Medicare and Medicaid Services and administration costs were estimated according to the Medicare Physician Fee schedule for 2018. The impact of grade 3 or 4 AEs was considered in the model.

### Sensitivity Analysis

To explore the uncertainty, one-way sensitivity analyses, probabilistic sensitivity analyses and scenario analyses were performed separately when using the mixture cure model or standard parametric model. The upper and lower limits of the inputs were derived from the original literature. For those inputs without upper and lower limits, the method of floating by 20% of the default input was used. In the probabilistic sensitivity analysis (PSA), we used visual basic programming to run 1,000 Monte Carlo simulations. Cost-effectiveness acceptability curves were then calculated.

For scenario 1, we assumed the utilities were from the other published literature, which were used in some other studies (11).

All parameters included in DSA/PSA and their variations are listed in Table 1.

**Table 1:** Key inputs for the partitioned survival model.

	**Deterministic**	**Distribution**	**Low**	**High**	**Source**
**QoL utility (per year)**					
PF	0.840	BETA	0.672	0.883	(23)
PP	0.473	BETA	0.166	0.568	
**Units for costs**					
Cost of atezolizumab/10 mg	77.46	GAMMA	77.01	77.76	(29)
Cost of etoposide/10 mg	0.66	GAMMA	0.56	0.74	
Cost of carboplatin/50 mg	3.09	GAMMA	2.89	3.25	
Cost of topotecan/0.1 mg	0.94	GAMMA	0.87	1.01	
Cost of cyclophosphamide/100 mg	3.91	GAMMA	3.31	4.54	
Cost of doxorubicin/10 mg	3.08	GAMMA	2.89	3.73	
Cost of vincristine/1 mg	4.88	GAMMA	4.79	5.00	
Supportive care cost	478	GAMMA	359	597	(9)
Death cost	9,433	GAMMA	7,075	11,791	
**Administration cost**					
First	144.72	GAMMA	115.78	173.66	(29)
Additional	31.68	GAMMA	25.34	38.02	
**Costs of MAEs**					
Neutropenia	17,181	GAMMA	13,745	20,617	(30)
Anemia	20,260	GAMMA	16,208	24,312	
Decreased neutrophil count	17,181	GAMMA	13,745	20,617	
Thrombocytopenia	22,698	GAMMA	20,289	25,377	
Leukopenia	17,181	GAMMA	13,745	20,617	
**Risk of AEs in A + C**					
Neutropenia	0.23	BETA	0.19	0.28	(5)
Anemia	0.14	BETA	0.11	0.17	
Decreased neutrophil count	0.14	BETA	0.11	0.17	
Thrombocytopenia	0.10	BETA	0.08	0.12	
Leukopenia	0.05	BETA	0.04	0.06	
**Risk of AEs in C**					
Neutropenia	0.25	BETA	0.20	0.29	(5)
Anemia	0.12	BETA	0.10	0.15	
Decreased neutrophil count	0.17	BETA	0.13	0.20	
Thrombocytopenia	0.08	BETA	0.06	0.09	
Leukopenia	0.04	BETA	0.03	0.05	
Discount rate	0.03	Fixed	0	0.05	(31)
**Disutilities of AEs**					
Neutropenia	−0.09	BETA	−0.122	−0.062	(23–25)
Anemia	−0.09	BETA	−0.133	−0.055	
Decreased neutrophil count	−0.09	BETA	−0.122	−0.062	
Thrombocytopenia	−0.108	BETA	−0.128	−0.089	
Leukopenia	−0.09	BETA	−0.122	−0.062	
**AEs duration**					
Neutropenia	2	Normal	2	2	(10)
Anemia	21	Normal	17	25	
Decreased neutrophil count	4	Normal	3	5	
Thrombocytopenia	24	Normal	19	28	
Leukopenia	2	Normal	2	2	

*A + C, atezolizumab + chemotherapy; C, chemotherapy; MAE, main adverse event; QoL, quality of life*.

### Ethics

The data of the PFS/OS curve was based on previously published trial, not from the database or the medical records. We used the Engauge Digitizer software to get the survival information from the figures in the paper and reconstructed data by ourselves, which was explained in the Section “Overall Survival and Progression-Free Survival.” Besides, the utilities and costs were derived from the published literatures, so ethics approval or specific consent procedures were not required for this study.

## Results

### Base-Case Results

In order to facilitate the identification of the models using the two extrapolation methods, we defined the model using the mixture cure model as model 1, and the model using the standard parametric model as model 2.

If the mixture cure model was considered for the intervention group, atezolizumab + chemotherapy was estimated to result in 0.73 QALYs, which was 0.11 QALYs higher than chemotherapy alone. In terms of LYGs, atezolizumab + chemotherapy was estimated to result in 1.12 LYGs, while chemotherapy alone was estimated to result in 1.1 LYGs. The total cost was higher in the atezolizumab + chemotherapy group, and atezolizumab + chemotherapy resulted in an average increase in patient lifetime costs of US$83,484. If the standard parametric model was considered for the intervention group, atezolizumab + chemotherapy was estimated to result in 0.73 QALYs, which was 0.15 QALYs higher than chemotherapy alone. In terms of LYGs, atezolizumab + chemotherapy was estimated to result in 1.12 LYGs, while chemotherapy alone was estimated to result in 1.11 LYGs. In comparing the results from the mixture cure model and standard parametric model, similar expected costs, expected outcomes and ICUR/ICER results could be seen by using either extrapolating approach. In the base-case analysis with a lifetime horizon, the costs and health outcomes calculated using the mixture cure model and standard parametric model are presented in Table 2.

**Table 2:** Results for the cost-effectiveness analysis in model 1 and model 2.

**Model**	**Group**	**Total Cost US$**	**Outcomes**	
			**Total QALYs**	**Total LYGs**
Model 1 (mixture cure model for A + C)	A + C	109,051	0.73	1.12
	C	25,556	0.63	0.96
	Incremental	83,484	0.11	0.16
	ICUR/ICER		US$785,848/QALY	US$529,888/LYG
Model 2 (standard parametric model for A+C)	A + C	109,824	0.73	1.11
	C	25,556	0.63	0.96
	Incremental	84,257	0.10	0.15
	ICUR/ICER		US$827,610/QALY	US$568,567/LYG

*A + C, atezolizumab + chemotherapy; C, chemotherapy*.

We found that the ICUR when using the standard parametric model was slightly higher than that when using the mixture cure model, with a difference of US$41,761/QALY. Similarly, the ICER when using the standard parametric model was slightly higher than that when using the mixture cure model, with a difference of US$38,679/LYG.

### Sensitivity Analysis

#### One-Way Sensitivity Analysis

**Figure 3:**
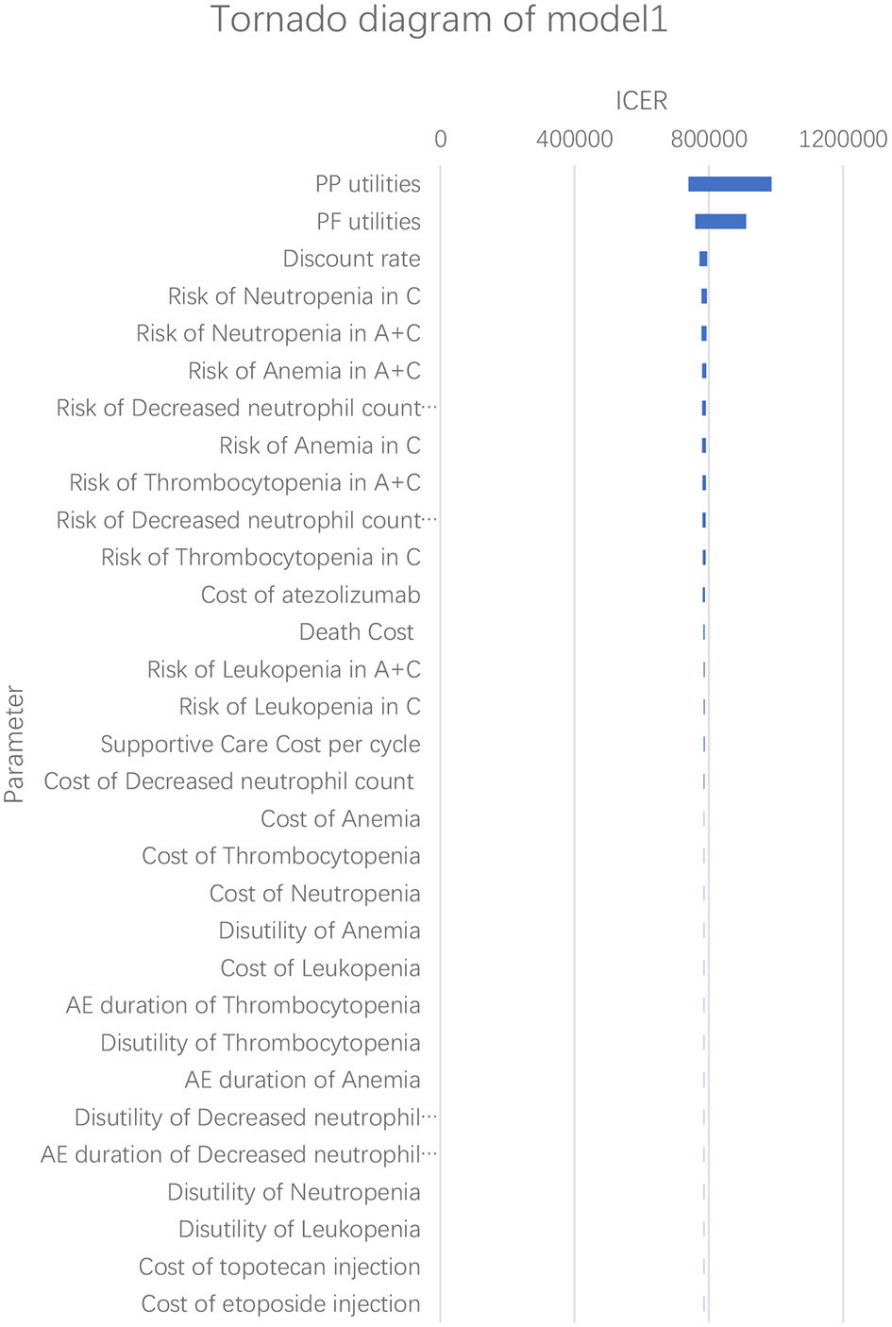
Tornado diagram of the one-way sensitivity analysis in model 1. A + C, atezolizumab + chemotherapy; C, chemotherapy.

**Figure 4:**
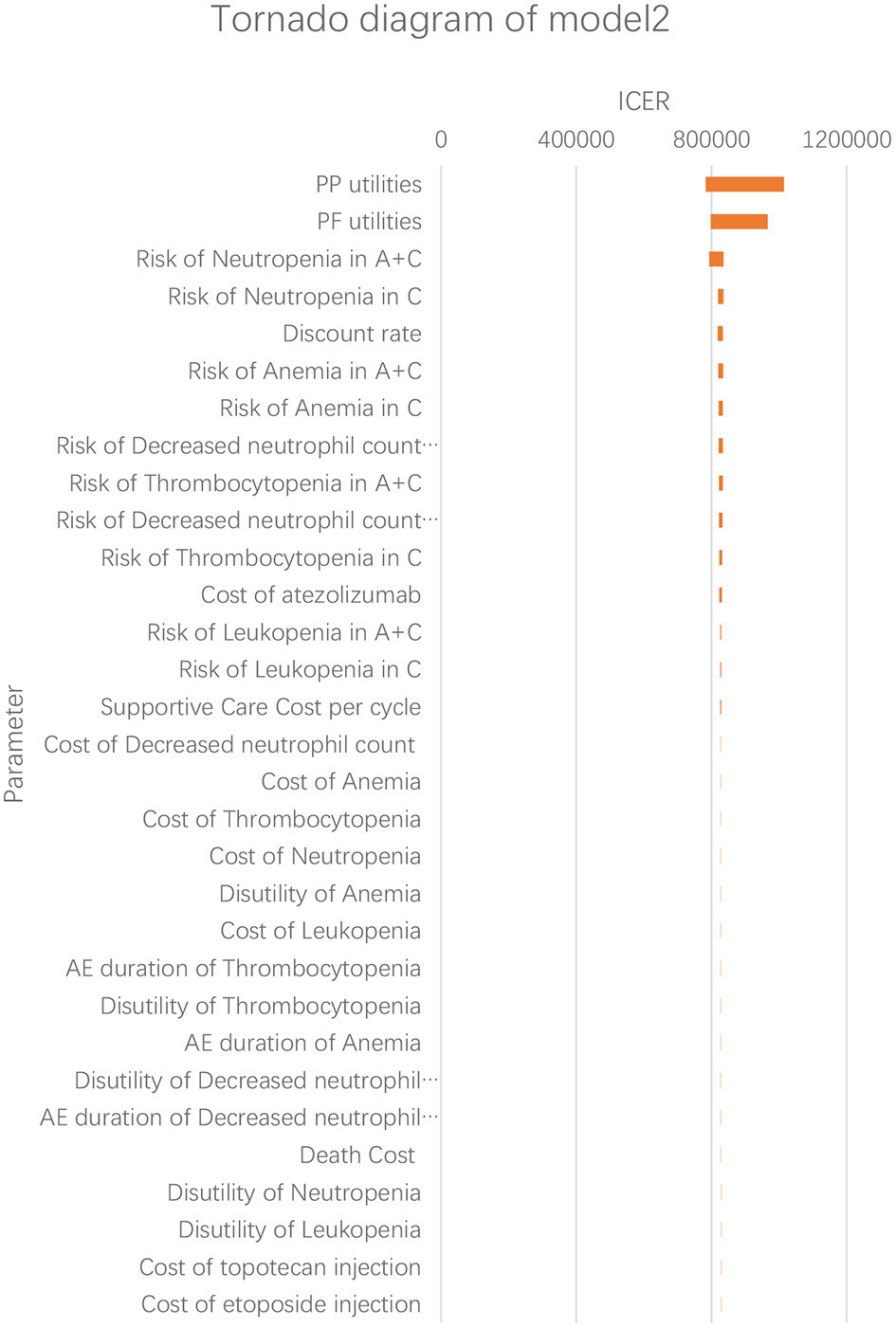
Tornado diagram of the one-way sensitivity analysis in model 2. A + C, atezolizumab + chemotherapy; C, chemotherapy.

According to the results of the one-way sensitivity analysis, we found that PP utility and PF utility were the main model drivers in both models (Figures 3, 4). The range of the one-way sensitivity analysis was from US$739,289/QALY to US$986,655/QALY in model 1, and the range of the one-way sensitivity analysis was from US$782,990/QALY to US$1,014,415/QALY in model 2.

#### Probabilistic Sensitivity Analysis

**Figure 5:**
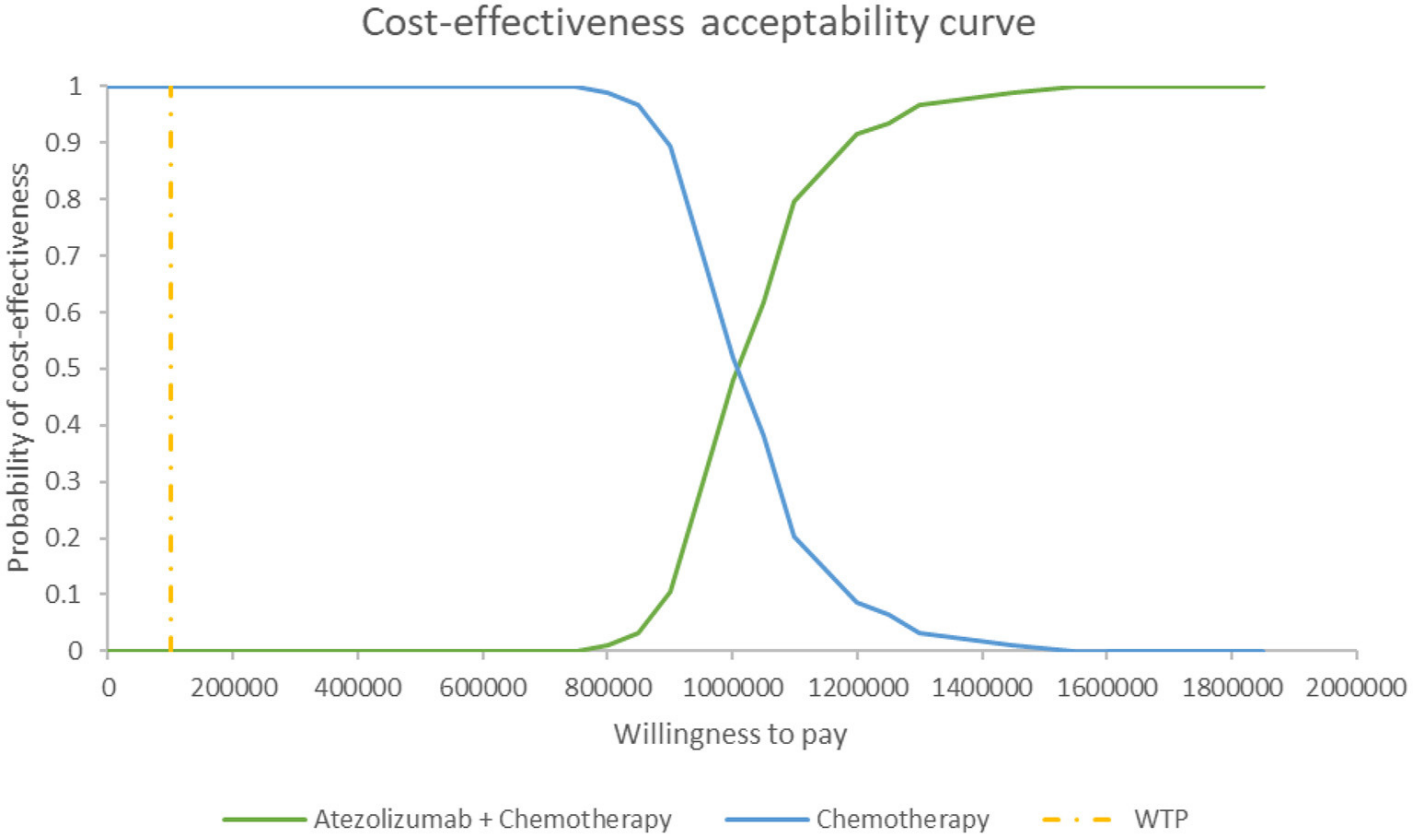
Cost-effectiveness acceptability curve at different thresholds of willingness to pay in model 1. WTP, willingness to pay.

**Figure 6:**
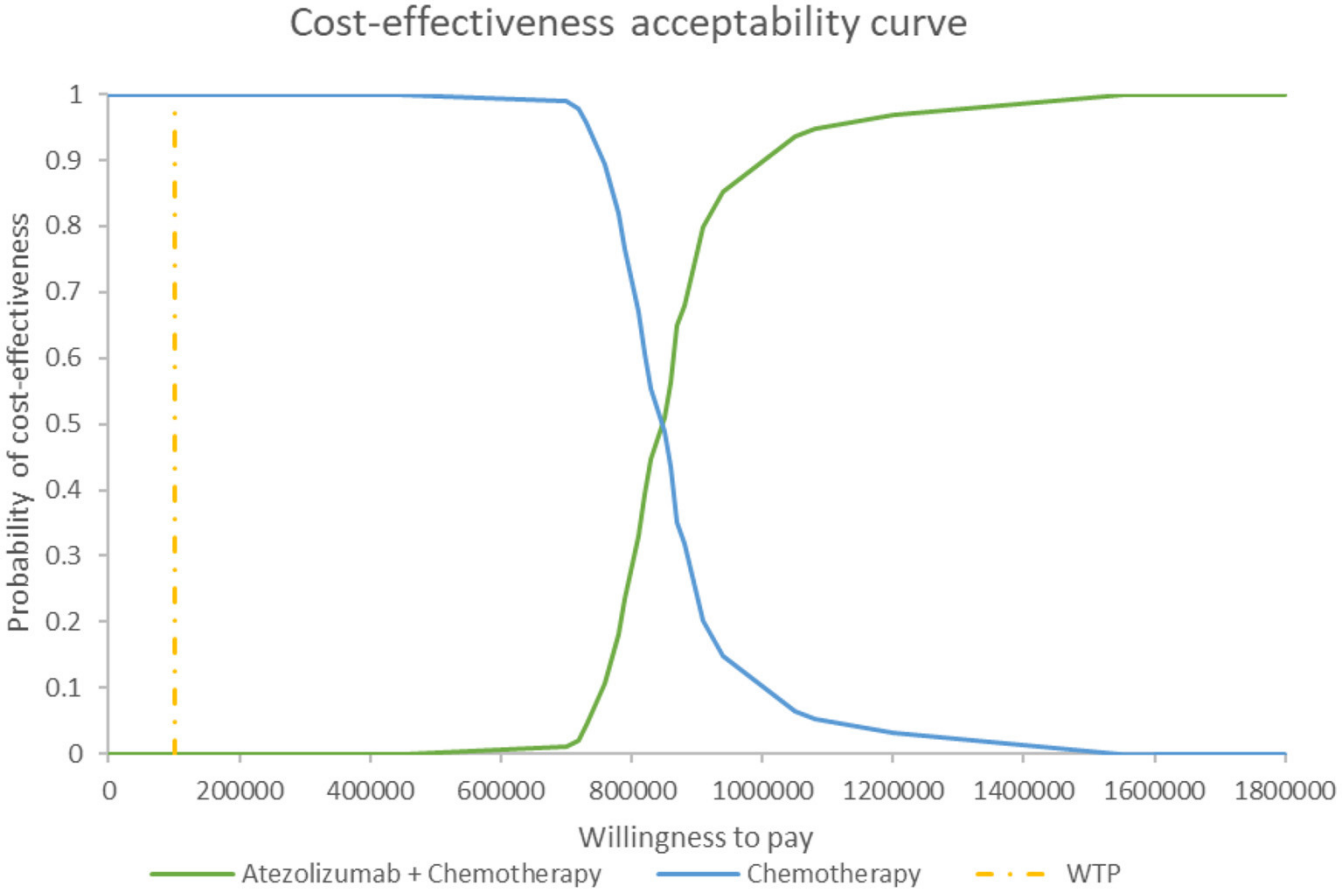
Cost-effectiveness acceptability curve at different thresholds of willingness to pay in model 2. WTP, willingness to pay.

Figure 5 shows the PSA results when the mixture cure model was used for the atezolizumab + chemotherapy group and the standard parametric model was used for the chemotherapy group. The cost-effectiveness threshold of US$100,000 per QALY was used as willingness to pay according to the recommendation of literature (32). The results demonstrated that atezolizumab + chemotherapy was associated with a 100% probability of being not cost-effective at the threshold of US$100,000 per QALY gained vs. chemotherapy alone. The same result was found when the standard parametric model was used for both the atezolizumab + chemotherapy group and the chemotherapy group (Figure 6).

#### Scenario Analysis

In the first scenario analysis in which we used the PF utility of 0.673 and the PP utility of 0.473, the result showed that the ICUR was US$910,557/QALY when the mixture cure model was used for the atezolizumab + chemotherapy group and the standard parametric model was used for the chemotherapy group. The ICUR was US$965,607/QALY when the standard parametric model was used for both the atezolizumab + chemotherapy group and the chemotherapy group.

## Discussion

This study evaluated the cost-effectiveness of atezolizumab + chemotherapy vs. chemotherapy alone in adult patients with SCLC from a US payer perspective based on data from the IMpower133 trial. The results of this economic evaluation indicated that atezolizumab + chemotherapy was more costly and more effective than chemotherapy alone for first-line treatment of extensive-stage SCLC. The estimated ICUR of US$785,848/QALY in model 1 and US$827,610/QALY in model 2 was much higher than the willing-to-pay threshold in US, which indicated that atezolizumab + chemotherapy was not cost-effective for first-line treatment of extensive-stage SCLC in US.

There existed one cost-effectiveness analysis based on IMpower133 trial data by Zhou K et.al. and we thought there were four deficiencies in that research. We supplemented and discussed these four points in this research: (1) That study only used exponential function for curve fitting, which could not reveal the delay effect in tumor immunotherapy. We used five standard parametric models and cure models to explore the most suitable fitting method. (2) The curve fitting did not discuss the potential long-term survival of tumor immunotherapy in that study, while we used the mixture cure model to assess the long-term uncertainty; (3) The previous literature only assumed the patients received the topotecan alone as second-line chemotherapy, which was inconsistent with the Impower133 experiment, while we used the follow-up therapy which was consistent with the Impower133 experiment; (4) PSA was not done in the previous literature, which is very important for the final conclusion and judgment, while we have done it.

For the special mechanism of PD-L1 and the obvious plateau at the end of the curve, the mixture cure model was also considered for the intervention group (carboplatin + etoposide + atezolizumab) to assess the long-term uncertainty, which was innovative to our best knowledge. It could be found that the intervention group would bring greater long-term survival benefits to patients when using the mixture cure model than when using the standard parametric model. Therefore, the total cost in model 1 was lower and the total QALYs and LYGs were higher and the ICER in model 1 was lower than that in model 2. We could see that when the willingness to pay was US$100,000/QALY, the results from both models demonstrated that atezolizumab + chemotherapy was not more cost-effective than chemotherapy alone in the US patients with SCLC.

Although there were certain differences in the results obtained by different extrapolation methods, it did not change the conclusion, which did not mean that the same conclusion would be reached using other data because it was associated with other factors, such as the length of the plateau period which might herald that there existed more “cured” patients.

Besides, difference in efficacy and safety of different chemotherapies would affect the results of this study. In the Impower133 experiment, the carboplatin + etoposide was used as the chemotherapy, but there are other chemotherapies such as cisplatin + etoposide, carboplatin + irinotecan and cisplatin + irinotecan which were recommended in NCCN Guidelines Small Cell Lung Cancer. Existing evidence shows that there is no huge difference in the efficacy and safety of these chemotherapy (33–36), but the irinotecan was much more expensive than other chemotherapy drugs. Therefore, if irinotecan was used as the chemotherapy, the results of this study might be more cost-effective.

In addition to atezolizumab, durvalumab was also recommended for the first-line treatment of extensive-SCLC according to NCCN Guidelines Small Cell Lung Cancer. It was found that one published literature explored the cost-effectiveness of durvalumab as a first-line treatment for extensive-SCLC https://doi.org/10.3389/fonc.2020.602185, which found that under a willingness-to-pay threshold of $100,000, durvalumab does not have a cost-effective comparative advantage in the US. Therefore, the existing evidence confirms that PD-L1 immunotherapies + chemotherapies were not cost-effective.

There were other shortcomings in our analyses. First, since the OS curve from the clinical trials did not meet the PH assumption, the non-mixture cure model could not be used, and it was impossible to detect the impact of the non-mixture cure model. According to the external data (21), we found that the standard parametric model was more authentic than the mixture cure model for the chemotherapy group. Therefore, for the chemotherapy group, we used a standard parametric model, which is different from the previous article. Due to the lack of external data about the atezolizumab + chemotherapy group, there was uncertainty in the exploration of the atezolizumab + chemotherapy group; therefore, we used two different methods to assess the uncertainty. Which model reflects the real treatment situation remains to be verified by long-term real-world data. Second, the utilities used in our study were not from the US SCLC patients. Therefore, we conducted scenario analysis and one-way sensitivity analysis to explore the uncertainty. Third, the adverse reactions included in the article were mainly grade 3 or higher adverse reactions with an incidence of more than 10% mentioned in clinical trials. However, clinical trials might not cover all the adverse reactions caused by atezolizumab + chemotherapy and chemotherapy alone due to the small sample size. In addition, because the dosages and usages we used were derived from clinical trials, there might be differences between clinical trials and real-world data. Therefore, the generalizability of study findings should be also a limitation of this study. Besides, many factors, especially PD-L1 expression might have a great impact on the effect of PD-1/L1 drugs. One published study revealed that when the expression of PD-L1 was different, PFS and OS of atezolizumab was obviously different for extensive-stage SCLC (37). It can be seen that when atezolizumab is used to treat SCLC patients with high PD-L1 expression, there exists an obvious plateau on the survival curves. Therefore, it might be more cost-effective for patients with high PD-L1 expression.

## Conclusions

With various approaches for estimating the relative effectiveness of atezolizumab + chemotherapy vs. chemotherapy alone, atezolizumab + chemotherapy was not found to be cost-effective compared with chemotherapy alone in adult patients with SCLC from a US payer perspective.

## Data Availability Statement

The original contributions presented in the study are included in the article/Supplementary Materials, further inquiries can be directed to the corresponding author/s.

## Ethics Statement

Ethical review and approval was not required for the study on human participants, in accordance with the local legislation and institutional requirements.

## Author Contributions

MR, AM, and HL contributed to the study conception and design. Material preparation and data collection were performed by YW, LY, XW, and MR, and analysis were performed by HL and AM. The first draft of the manuscript was written by MR and YS. All authors commented on subsequent versions of the manuscript and read and approved the final manuscript.

## Conflict of Interest

The authors declare that the research was conducted in the absence of any commercial or financial relationships that could be construed as a potential conflict of interest.
