# Supplementary material for: Economic Evaluation of First-Line Atezolizumab for Extensive-Stage Small-Cell Lung Cancer in the US
Source: Front Public Health. 2021 Apr 6;9:650392. doi: 10.3389/fpubh.2021.650392 (PMC8055835; doi:10.3389/fpubh.2021.650392)
Supplement: Supplementary file 1 [file Data_Sheet_1.docx]

# Supplementary Material

Economic Evaluation of First-Line Atezolizumab for Extensive-Stage Small-Cell Lung Cancer in the U.S.

# Estimation of Parametric Survival Distributions for Overall Survival and Progression-Free Survival

Table S1 Results of fitting to the observed data

|  |  | Exponential | Weibull | Gompertz | Log-logistic | Log normal |
| --- | --- | --- | --- | --- | --- | --- |
| PFS A+C | AIC | 1280 | 1243 | 1269 | 1243 | 1216 |
| PFS C | AIC | 1188 | 1121 | 1163 | 1145 | 1092 |
| OS A+C | AIC | 1352 | 1299 | 1296 | 1357 | 1317 |
| OS C | AIC | 1356 | 1321 | 1309 | 1344 | 1316 |
| Mixture cure model |  | Exponential | Weibull | Gompertz | Log-logistic | Log normal |
| OS A+C | AIC | 2713 | 2610 | 2603 | 2705 | 2638 |

A+C: atezolizumab + chemotherapy; C: chemotherapy; AIC: Akaike information criterion; PFS: progression-free survival; OS: overall survival

Table S2 Best fitting and the value of the parameter

|  | Fitting | λ/μ | γ/σ | Cure fraction |
| --- | --- | --- | --- | --- |
| C PFS | Lognormal(μ, σ) | 1.7725 | 0.7262 |  |
| C OS | Gompertz(λ, γ) | 0.0294 | 0.06022 |  |
| A+C PFS | Lognormal(μ, σ) | 1.9174 | 0.7771 |  |
| A+C OS | Gompertz(λ, γ) | 0.01845 | 0.0767 |  |
|  | Mixture cure+ Gompertz (restricted) | 0.02051 | 0.09015 | 0.11433 |

A+C: atezolizumab + chemotherapy; C: chemotherapy; PFS: progression-free survival; OS: overall survival
